# Point-of-Care Ultrasound Protocol for Insertion and Confirmation of Central Venous Catheter Placement

**DOI:** 10.7759/cureus.29259

**Published:** 2022-09-17

**Authors:** Pedro R Soares, Andre Maia, Jorge R Fernandes, Diogo Faustino, Ana Luísa Campos, Luís R Almeida, José Mariz

**Affiliations:** 1 Internal Medicine, Centro Hospitalar Universitario Sao Joao, Porto, PRT; 2 Internal Medicine, Hospital Vila Real, Vila Real, PRT; 3 Internal Medicine, Centro Hospitalar Universitário de Lisboa Central, Lisbon, PRT; 4 Internal Medicine, Centro Hospitalar Lisboa Central, Hospital São José, Lisbon, PRT; 5 Internal Medicine, Hospital da Senhora da Oliveira, Guimarães, PRT; 6 Emergency Department, Hospital de Braga, Braga, PRT

**Keywords:** point-of-care ultrasound, cvc confirmation, pocus, ultrasound, central venous catheter, vascular access

## Abstract

Central venous catheterization is a common procedure in the management of critically ill patients, in the context of medical emergencies, and before surgical interventions. Placing a central venous catheter (CVC) in the internal jugular vein (IJV) using anatomical references is associated with a high risk of complications, in particular pneumothorax and arterial puncture. Thus, the placement of CVCs with ultrasound support is recommended by several medical societies and health regulators at the international level. When compared with chest radiography, ultrasound is accessible, safe, cost-effective, and time efficient. This technical report is meant to detail a point-of-care ultrasound protocol designed for the insertion and confirmation of the correct placement of a CVC in the IJV.

## Introduction

Central venous catheterization is a common procedure that is frequently required during the treatment of surgical and/or critically ill patients. The placement of a central venous catheter (CVC) in the internal jugular vein (IJV) using anatomical references alone is associated with a high risk of complications, in particular pneumothorax and undue arterial puncture, with a reported incidence as high as 19% [1,2].

The placement of a CVC with ultrasound guidance is an accessible and safe method, increasingly highlighted by the medical community [3,4]. This technical report proposes a point-of-care ultrasound (POCUS) protocol designed for the insertion and confirmation of the correct placement of a CVC in the IJV.

## Technical report

POCUS protocol for CVC insertion

Preprocedural scanning of the left and right neck should be performed to determine the best CVC insertion site. A linear transducer is placed transversely on the anterolateral neck and the IJV is assessed from the angle of the jaw to the inferior anastomosis with the subclavian vein. The IJV's size, shape, depth, compressibility, and proximity to the carotid artery (CA) should be evaluated; it is particularly important to assess for complete compressibility of the IJV as asymptomatic thrombosis may occur [4]. The safest needle insertion site should be based on the following characteristics: absence of thrombus, widest diameter, shallowest depth, and relationship to the CA (preferably not overlying) [4].

The placement of a CVC in the IJV with a POCUS protocol requires the use of an ultrasound probe throughout the procedure until the puncture [3]. After surgical hand washing, placement of sterile equipment (coat and gloves), extensive disinfection of the puncture site (with povidone-iodine or 2% chlorhexidine), placing sterile drapes or plastic sheaths, confirming the functioning and filling of the various pathways of the CVC, and the administration of local anesthesia, the probe is placed inside the sterile sleeve with gel. If possible, the patient should be placed in the Trendelenburg position with a 15º depression to increase the IJV lumen [5,6].

**Figure 1:**
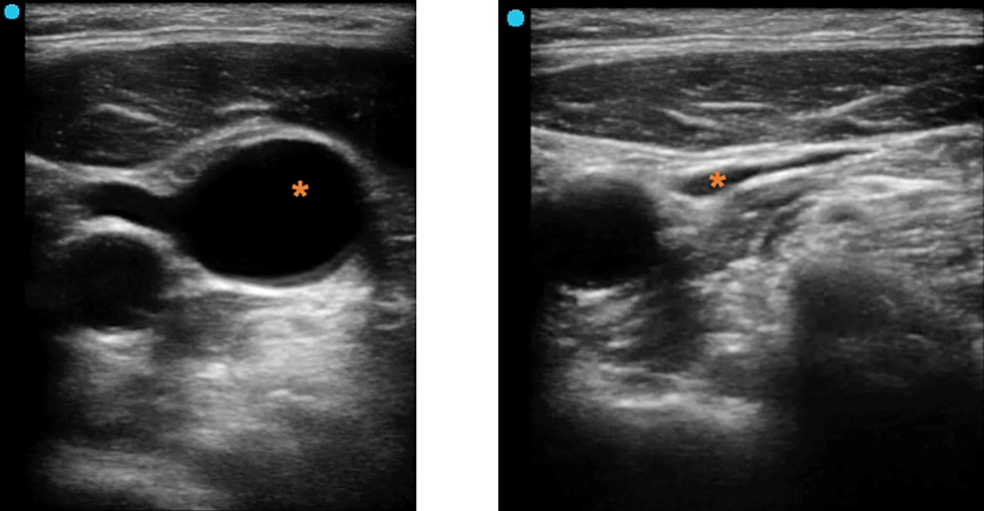
Sonoanatomy of the IJV (*) and its topographical relationship with CA. On the right, the compressibility of the vein compared to the artery is demonstrated. Sonoanatomy of the right IJV with the operator at the head of the bed and the indicator probe point directed to the patient's left side. IJV: internal jugular vein; CA: carotid artery.

The manipulation of the probe should be performed with the non-dominant hand. After correctly identifying the IJV and the CA, the ultrasound probe should be placed perpendicularly (making an angle that should be close to 90º with the skin, to obtain a transverse image). The use of the probe in this position allows the continuous visualization of the IJV, the CA, and their topographic relation. Once the IJV has been identified (Figure 1), the image must be centered prior to needle introduction. The needle should be introduced in continuous aspiration at an angle of approximately 60º in relation to the skin.

For the longitudinal approach to the IJV, the probe must be rotated by 90º. The image obtained allows better visualization of the needle entry into the IJV, in relation to the transverse approach. The angle between the needle and the skin should be 30-45º.

Once the blood is aspirated in the syringe, the angle between the needle and the skin should be slightly lowered prior to guidewire insertion. Once the guidewire is inserted through the needle, the operator should continue the process according to the Seldinger technique (recommended electrocardiographic monitoring; if there are any cardiac disturbances, such as atrial arrhythmias, ventricular ectopics, or heart block, the guidewire should be slightly withdrawn). The ultrasound (US) can be used to confirm the guidewire positioning inside the IJV before using the dilator and inserting the CVC.

POCUS protocol for CVC placement confirmation

**Figure 2:**
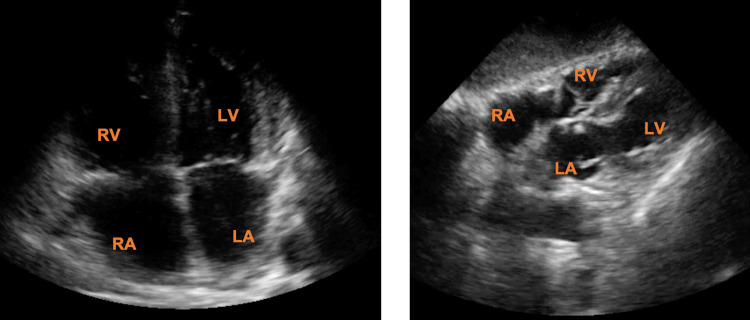
Apical four-chamber (left) and subcostal four-chamber (right) windows in two-dimensional mode. RA: right atrium; LA: left atrium; RV: right ventricle; LV: left ventricle.

The following confirmation protocol can be used to confirm the correct placement of any supradiaphragmatic CVC. With the patient in the supine position and with a low-frequency cardiac sector probe, echocardiographic windows (apical four-chambers and/or subcostal bicaval view) should be obtained for correct visualization of the right atrium (RA) (Figure 2).

**Figure 3:**
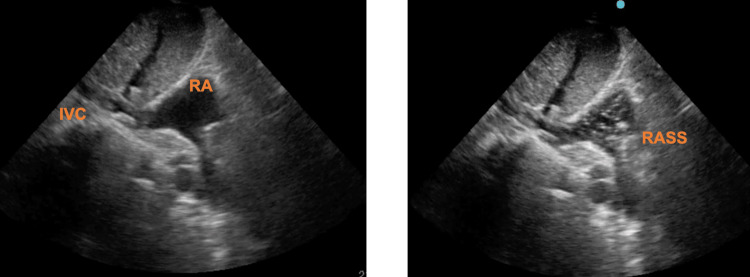
Subcostal bicaval view visualization of a turbulent flow in the right atrium (RA), after the administration of 5 mL of a saline solution in the CVC. CVC: central venous catheter; IVC: inferior vena cava; RASS: rapid atrial swirl sign.

Then, 5 milliliters (mL) of sterile saline solution should be administered in one of the CVC lumens, preferably through the lumen opening at the tip. The CVC is considered to be correctly placed if a rapid atrial swirl sign (RASS) is visualized (Figure 3) [7,8].

Exclusion of pneumothorax

Pneumothorax is a potential mechanical complication after CVC placement and it may be detected hours to a couple of days after the procedure. The clinical scenario and physical examination dictate the timing of evaluation and a pneumothorax should be suspected in a new or worsening dyspnea or pleuritic chest pain after a CVC placement.

**Figure 4:**
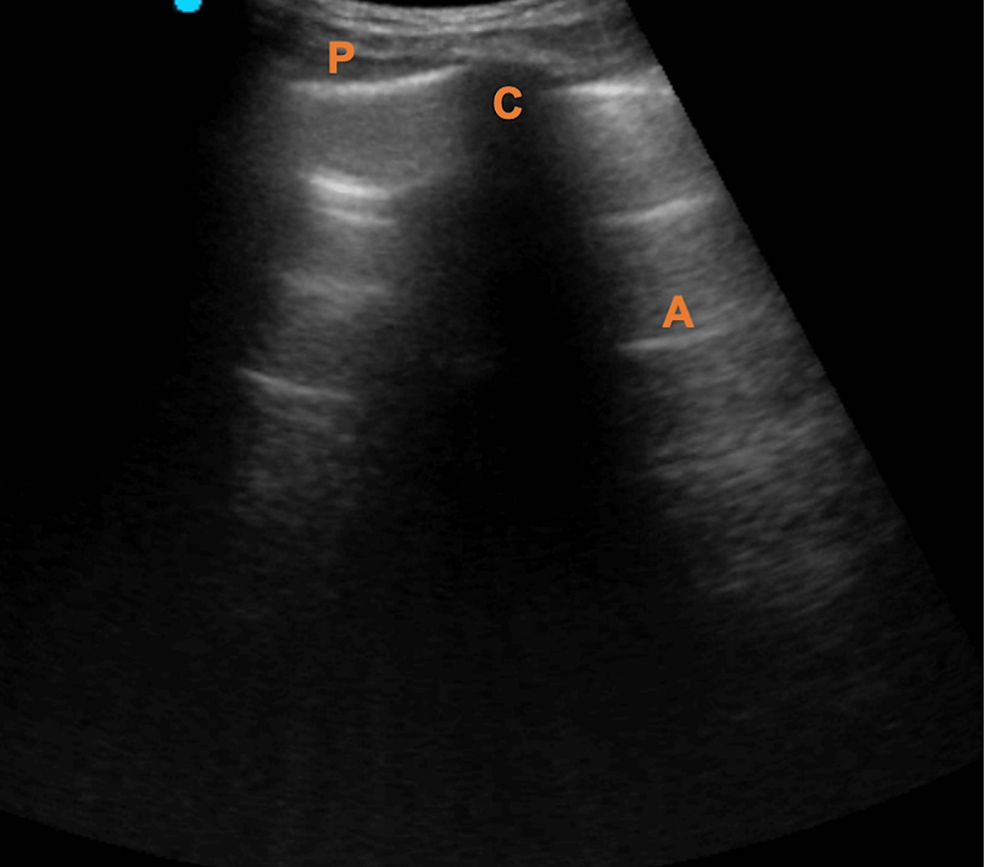
Assessment of the left apex position using the lung ultrasound. P: pleura; A: A lines; C: rib (and acoustic shadow).

This step is performed with the patient in the supine position and with a high-frequency linear probe (or alternatively with a curvilinear or sectoral probe). The probe must be placed in a sagittal position on the anterior surface of the thorax, close to the second intercostal space, in the midclavicular line. Sonographic references include the presence of two ribs with their respective posterior acoustic shadow and the pleural line between them (Figure 4).

**Figure 5:**
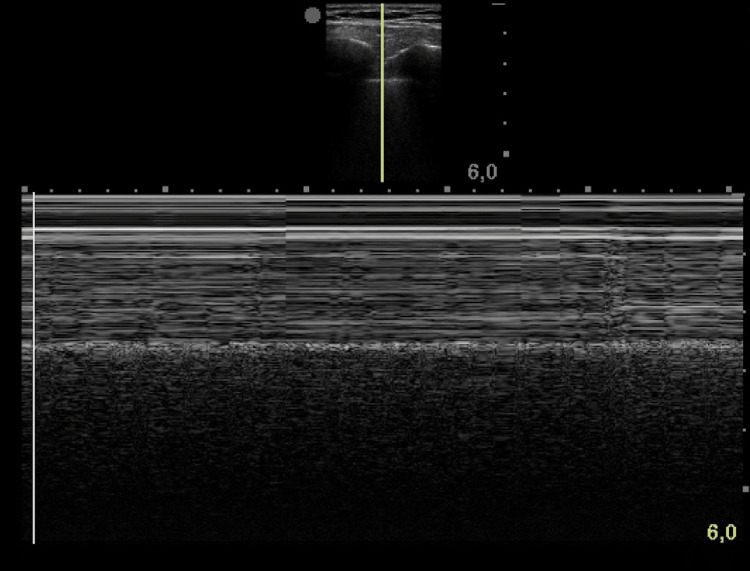
The observation of the seashore sign using the M-mode can be useful to rule out procedure-related pneumothorax.

The detection of lung sliding is the most important finding for confirmation of the presence of an adequately ventilated lung, and it must be done before and after the CVC placement. The use of M-mode (Figure 5) allows the evaluation of the relationship between movement and time, which may be particularly useful in patients whose lung sliding is particularly subtle (including the elderly or in the case of low lung reserve). The evaluation of the pulmonary parenchyma should be made by interrogating the anterior wall. The absence of lung sliding and detection of the lung point allows the sonographic diagnosis of pneumothorax.

## Discussion

CVC placement is a regularly performed procedure in the intensive care unit (ICU), emergency department (ED), and in anesthesia induction area. Previous studies have shown that ultrasound guidance improves patient safety and CVC placement performance in the IJV [9]. Based on compounding evidence from previous clinical studies, increasing medical societies worldwide recommend the use of a POCUS protocol for CVC placement in the IJV [9].

Despite being noninvasive and not bearing risk to the patient, US has some limitations and disadvantages that should be considered. An insufficient number of US machines in an ICU or ED may cause procedural delays; moreover, the purchase and maintenance of US machines and adequate training for all operators involved in CVC placement may be expensive [10]. US may be associated with a false sense of security to an inexperienced user, misleading to neglect traditionally taught principles with regard to needle direction [9]. According to Nandy et al., as many as 30 cannulations need to be observed and performed under an ultrasound-guided technique to achieve competency [11]. To overcome these problems related to US skills and ensure optimal care, training and simulation of US skills for vascular access are necessary [12]. The application of a POCUS protocol during CVC placement may be a valuable tool to improve safety and standardize care.

US has an important role in the confirmation of CVC correct placement and to evaluate for postprocedural complications. Traditionally, to ensure the correct placement of the CVC and to exclude complications, including pneumothorax, a chest radiograph is usually ordered. When compared to this method, POCUS has several advantages, which include its lack of radiation, speed, the requirement of only one provider, and avoiding the need to move the patient for imaging. A previous study revealed that POCUS confirmation of CVC placement was on average 24 minutes faster than chest radiography [8]. Additionally, the use of US has an overall sensitivity and specificity of 96% and 100% to assure the correct placement of a supradiaphragmatic CVC [7,13]. When compared to chest radiography, POCUS confirmation of CVC positioning may be superior as the catheter will project over the aorta or lung while lying within the venous system [14]. Although a limitation of POCUS is its user dependence and that performing a bubble study evaluation through echocardiography is not part of standard training, the technique is easily adapted for those with little experience [15].

In a study by Duran-Gehring et al., POCUS edified all complications associated with CVC placement [8]. Additionally, Amir et al. demonstrated that POCUS is non-inferior to chest radiography when used to screen for postprocedural pneumothorax [16]. The sonographic detection of lung sliding allows the exclusion of pneumothorax in the site examined with a sensitivity and specificity of near 100% [3], making POCUS a valuable tool not only for the confirmation of correct positioning within the venous system but also to exclude potential procedural complications.

## Conclusions

The use of a POCUS protocol for the verification of CVC correct placement is a radiation-free method that also allows for faster confirmation in comparison to chest radiography, which may be particularly beneficial when treating patients in the ED or the ICU. Additionally, the use of US for exclusion of procedure-related complications, particularly pneumothorax, is cost-effective and possesses high sensitivity and specificity when compared to traditional methods. Although POCUS may be user-dependent, the RASS evaluation is easily adapted for those with little to no experience. A POCUS protocol for CVC insertion, confirmation of correct positioning in the venous system, and exclusion of procedural complications promotes patient safety and the optimal use of resources while saving time.
